# Trochanteric Femur Fractures: Application of Skeletal Traction during Surgery Does Not Alter Soft-Tissue Microcirculation

**DOI:** 10.3390/medicina57090884

**Published:** 2021-08-27

**Authors:** Kenneth P. van Knegsel, Bergita Ganse, Pascal C. Haefeli, Filippo Migliorini, Mario F. Scaglioni, Bryan J. M. van de Wall, Bong-Sung Kim, Björn-Christian Link, Frank J. P. Beeres, Sven Nebelung, Carsten Schoeneberg, Frank Hildebrand, Boyko Gueorguiev, Matthias Knobe

**Affiliations:** 1 Department of Orthopaedic and Trauma Surgery, Cantonal Hospital of Lucerne, Spitalstraβe 16, 6000 Lucerne, Switzerland; pascal.haefeli@luks.ch (P.C.H.); byan.vandewall@luks.ch (B.J.M.v.d.W.); bjoern-christian.link@luks.ch (B.-C.L.); frank.beeres@luks.ch (F.J.P.B.); matthias.knobe@luks.ch (M.K.); 2 AO Research Institute Davos, Clavadelerstrasse 8, 7270 Davos Platz, Switzerland; boyko.gueorguiev@aofoundation.org; 3 Werner Siemens Foundation Endowed Chair for Innovative Implant Development, Clinics and Institutes of Surgery, Saarland University, 66421 Homburg, Germany; bergita.ganse@uks.eu; 4 Department of Trauma, Hand and Reconstructive Surgery, Saarland University Hospital, 66421 Homburg, Germany; 5 Department of Orthopaedic Surgery, University Hospital RWTH Aachen, Pauwelsstrasse 30, 52074 Aachen, Germany; fmigliorini@ukaachen.de (F.M.); fhildebrand@ukaachen.de (F.H.); 6 Department of Plastic and Hand Surgery, Cantonal Hospital of Lucerne, Spitalstraβe 16, 6000 Lucerne, Switzerland; mario.scaglioni@luks.ch; 7 Department of Plastic and Hand Surgery, University Hospital Zürich, Rämistraße 100, 8091 Zürich, Switzerland; bong-sung.kim@usz.ch; 8 Department of Radiology, University Hospital RWTH Aachen, Pauwelsstrasse 30, 52074 Aachen, Germany; snebelung@ukaachen.de; 9 Department of Orthopaedic and Emergency Surgery, Alfried Krupp Hospital, 45131 Essen, Germany; carsten.schoeneberg@krupp-krankenhaus.de

**Keywords:** microcirculation, traction table, traction, trochanteric, femur, fracture, blood flow, saturation, hemoglobin, laser-Doppler spectrophotometry, soft-tissue

## Abstract

*Background and Objectives*: Wound infections provoked by alterations in microcirculation are major complications in the treatment of trochanteric femur fractures. Surgical fracture fixation on a traction table is the gold standard for treatment, but the effect on tissue microcirculation is unknown. Microcirculation could be impaired by the pull on the soft-tissue or by a release of vasoactive factors. We hypothesized that intraoperative traction impairs soft-tissue microcirculation. *Materials and Methods*: In 22 patients (14 women, eight men), average age 78 years (range 36–96 ± 14), with trochanteric femur fractures, non-invasive laser-Doppler spectrophotometry was used to assess oxygen saturation, hemoglobin content, and blood flow in the skin and subcutaneous tissue before and after application of traction. Measurements were recorded in nine locations around the greater trochanter at a depth of 2, 8, and 15 mm before and after fracture reduction by traction. *Results*: No differences were found in any depth with traction compared to without (oxygen saturation: *p =* 0.751, *p =* 0.308, and *p =* 0.955, haemoglobin content: *p =* 0.651, *p =* 0.928, and *p =* 0.926, blood flow: *p =* 0.829, *p =* 0.866, and *p =* 0.411). *Conclusion*: In this pilot study, the application of traction does not affect skin and subcutaneous microcirculation in the surgery of proximal femur fractures.

## 1. Introduction

Proximal femur fractures are among the most common fractures worldwide and have a major impact on healthcare systems [1,2]. Despite multiple treatment options, improved implants, advanced research and knowledge, the two year mortality rate in these old and often multi-morbid patients is still between 9 and 43% [1,3,4,5]. Among factors relevant to the high mortality rate are post-operative complications, such as surgical-site infections, nonunion, deep vein thrombosis, and increased morbidity [1,6,7]. Closed reduction and internal fixation (CRIF) is the gold standard in the treatment of trochanteric femur fractures, resulting in shorter hospital stays, reduced costs, and better anatomical reduction compared to the non-operative six week in-bed traction treatment [6,8]. In some countries, skeletal or skin traction is still used pre-operatively to cover the time until surgery, even though a Cochrane review concluded no benefit [9,10].

**Figure 1:**
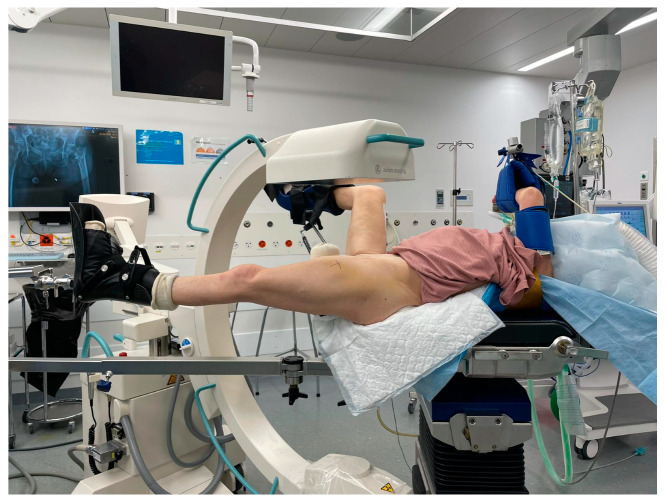
Setup in the operating theatre with skeletal traction applied to position the patient and reduce the fracture under fluoroscopic guidance.

A traction table is commonly used to perform and hold the reduction in the fracture during surgery (Figure 1) [11,12]. Although traction tables have an advantage during the operation, due to the strong forces applied, they do not come without complications, including soft-tissue damage, neurologic impairment, and iatrogenic compartment syndrome [11]. Furthermore, microcirculation could potentially be impaired by the pull on the soft-tissue and compression of blood vessels, as well as by a release of vasoactive factors from the fracture site.

Ganse et al. [13] published the first study to measure and show changes in microcirculation after CRIF of trochanteric femur fractures and their relation to risk factors, and the type of implant. Significant differences in the parameters of microcirculation were found comparing the healthy and the injured leg, as well as throughout the course of the post-operative period [13]. Microcirculation plays an important role in keeping the local environment optimal for tissue healing. Decreases in blood supply and venous outflow diminish oxygen supply and cause hypoxia, acidosis, collection of metabolites, and oxidative/nitroxidative stress and, subsequently, lead to poor tissue healing, impairment of the immune system, and possibly even cell death and necrosis [13,14,15]. Impairment of the local immune system promotes bacterial growth and inflammation [14]. In summary, impaired microcirculation before and after surgery can have a negative impact on the healing process and promote complications.

No data have ever been published that show the effect of skeletal traction on skin and subcutaneous microcirculation in human fracture patients. Therefore, we hypothesized that intraoperative traction impairs soft-tissue microcirculation around the greater trochanter in the surgery of trochanteric femur fractures, and conducted a first pilot study.

## 2. Materials and Methods

The study was carried out in accordance with the recommendations of the Committee on Publication Ethics (COPE) and the International Committee of Medical Journal Editors (ICMJE). The University Hospital IRB (reference number EK006/11, date of approval: 15 April 2011) approved the study protocol. This prospective cohort study was registered on www.ClinicalTrials.gov (number NCT01264172, date 21 July 2010) with the title “Identification of Microcirculation and Inflammation after Minimally-invasive Osteosynthesis of the Proximal Femur” (MicroProxFem) [13]. Written informed consent was collected in accordance with the Declaration of Helsinki.

### 2.1. Study Design

The inclusion criterion was presentation to the emergency room of a level 1 trauma center with a traumatic trochanteric femur fracture. The exclusion criteria were polytrauma, suspected pathological fractures, open fractures or severe soft-tissue damage, fractures extending more than 5 cm distal to the lesser trochanter, a known history of metabolic bone diseases, prior surgery of the hip or femur, leg deformities, and immunodeficiency. Patients were also excluded if the surgical intervention was performed later than 4 days after the accident, independent of the underlying cause. All patients were admitted and kept in supine position until surgery without skeletal traction. Traction was then applied on the operation table in all patients with the minimal force needed to reduce the fracture and thereby minimize the risk of potential complications. Skeletal traction involves axial, shear, and rotational components (Figure 1). Varying levels of traction and manual manipulation of the leg are needed to reduce and keep the fracture in place. The amount of force needed depends on the fracture morphology, body mass, and muscle tone. To avoid injury, only the minimum necessary force is applied. Due to the many confounding factors, such as direction, patient weight, fracture morphology, angle of rotation, etc., the applied forced cannot be properly quantified.

### 2.2. Measurements

**Figure 2:**
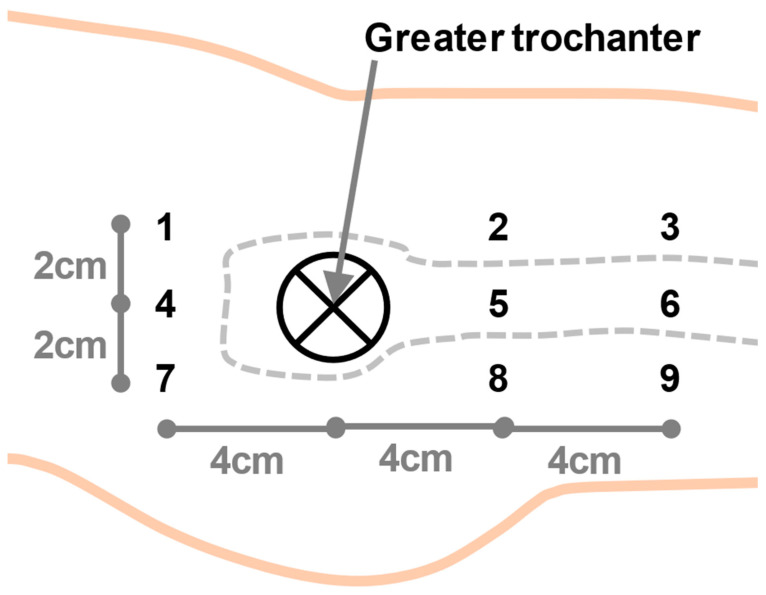
The greater trochanter was used as a landmark to locate the measurement points 1–9.

Measurements were taken immediately before and after fracture reduction. The procedure of fracture reduction differs in time span, usually varying between 2 and 10 min. A non-invasive Laser-Doppler spectrophotometry system (“Oxygen to see,” O2C, LEA Medizintechnik, Winchesterstr. 2, D-35394 Gießen, Germany) was used to assess three separate parameters that reflect microcirculation. The white-light spectrum range (500–800 nm) was detected with a continuous laser light (830 nm and 30 mW) through an LH-2 fibreglass probe on the patients’ skin. The oxygen saturation (SO_2_) was computed by the O2C device from the change in color of the reflected light. The change is caused by partial light absorption through the hemoglobin. The amount of absorption is utilized to calculate the relative hemoglobin amount (Hb). The blood flow (flow) in the capillary system is measured via the detected laser-Doppler shift [13,16]. Measurement values were separately recorded for 2, 8, and 15 mm depths. Two measurements were taken in the operation theatre before (t0) and after (t1) fracture reduction under traction. The probe was temporarily affixed on the patients’ skin using tape to standardize contact pressure and left in place for both measurements. Measurements were taken in all three depths at nine different locations surrounding the greater trochanter, as depicted in Figure 2. All patients were without catecholamine medication during the measurements. The data of all measurement points were pooled for the analysis. Measurements were only taken from the injured leg and not from the healthy leg, as skeletal traction is only applied to reduce the fracture on the injured side. Traction is associated with a risk of complications and therefore only applied to the injured leg. The room temperature was kept constant at 21 °C. The patient’s body temperature was kept in the physiological range while being monitored by the anesthesiologist.

### 2.3. Statistical Analyses

Statistical analysis was performed using the SPSS software package (IBM SPSS Statistics release 21.0.0, Armonk, NY, USA). Flow values greater than 1000 arbitrary units (AU) were considered out of bounds and excluded from analysis. Normal distribution of the data was tested by the Shapiro–Wilk Test. After paired data transformation by 10log, x^2^, x^4^, 1/x, and square root, the data were normally distributed. One-way ANOVA was used to analyze the effect of traction between the time points t0 and t1.

## 3. Results

**Figure 3:**
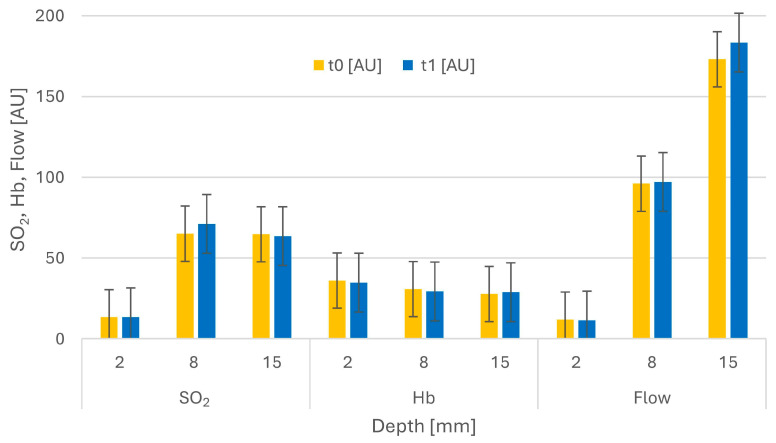
Mean values of t0 and t1 in the three depths in arbitrary units (AU). Bars indicate SD.

In total, 22 patients (14 women and eight men) with a trochanteric femur fracture were included. The average age was 78 years (range 36 to 96 ± 14). Four of the patients had type 2 diabetes mellitus and three smoked. In all patients, fracture reduction and the operation were performed on the traction table. None of the patients had delayed wound healing or needed revision surgery. In nine patients, the flow rate at 15 mm depth was greater than 1000 AU, and these data were therefore treated as outliers. Table 1 and Figure 3 show the study results in detail.

There were no significant differences in SO_2_ before and after the application of traction in any of the three depths (2 mm *p* = 0.751, 8 mm *p* = 0.308, and 15 mm *p* = 0.955). There was no significant difference in Hb before and after the application of traction in all three depths (*p* = 0.651, *p* = 0.928, and *p* = 0.926). No significant difference in flow before and after the application of traction was found in any of the three depths (*p* = 0.829, *p* = 0.866, and *p* = 0.411, Table 1).

## 4. Discussion

The findings of the present study indicate no differences in microcirculation (SO_2_, Hb and Flow) in any of the measured depths (2, 8, and 15 mm) comparing before and after the application of skeletal traction. Therefore, traction does not compromise soft-tissue microcirculation in patients undergoing surgery for proximal femur fractures.

Ganse et al. showed that, in proximal femur fractures, the surgery itself alters skin and subcutaneous microcirculation around the greater trochanter throughout the post-operative period, compared to the healthy leg. The same was previously demonstrated for calcaneal and thoracolumbar fractures, where microcirculation, in a minimally invasive approach, was compared to standard open fixation [17,18]. It is a common observation that microcirculation is altered after trauma [15,19]. In addition, it is known that short-term changes in microcirculation can be induced by the intervention of low-intensity pulsed ultrasound (LIPUS) [20].

An inhibition of local microcirculation leads to decreases in oxygen supply, the development of hypoxia, acidosis, and a collection of metabolites, causing oxidative and nitroxidative stress [14,15]. The local capillarization is determined by factors such as local substrate delivery and metabolite removal [21]. Induced impairments in the function of the immune system support bacterial growth [14]. The differences in oxygen saturation, hemoglobin, and blood flow between the different skin and subcutaneous tissue depths, as shown in Figure 3, were not the focus of this study, as they are physiological and can be explained by the anatomy and physiology of skin microcirculation [13,22].

The application of traction reverses the shortening and malrotation of the leg by reducing the fracture and by countering muscle tension, which is necessary to successfully treat the fracture, but does not come without complications [11,12]. Known complications of skeletal traction include soft-tissue damage, neurologic impairment, and iatrogenic compartment syndrome [11]. These complications not only correlate with the force applied by the traction table, but also seem to induce structural changes in the soft-tissue environment, i.e., reductions in microcirculation after the application of traction. Fracture reduction recreates the normal anatomical alignment, which might be beneficial for microcirculation. This is the reason why, in some countries, skeletal or skin traction are used pre-operatively when surgery cannot be conducted immediately [9,10].

In the present study, the data were collected before the operation and after applying traction using the traction table. The time between the application of traction and the measurement with traction was therefore relatively short. Prolonged surgery times with traction and the application of pre-operative traction might affect microcirculation more substantially, but this was not the focus of the present analysis. In addition, possible alteration in the course of microcirculation throughout the mid-term post-operative period should be addressed in future studies.

There were a few limitations to this study. A large standard deviation of the data was observed, which is in line with the known high inter-individual variability in microcirculation [16,23]. In addition, the number of patients in the present study was too low to assess differences between sexes and age-effects. Of note, this pilot study aimed at finding differences in microcirculation with and without traction, while sex- and age-effects were not part of the hypothesis. The assessment of group differences (e.g., age, gender, smoker status, diabetes status, etc.) in larger patient collectives is an interesting topic for subsequent studies. Effects of vasoactive substances and anesthetic agents, such as catecholamines, on soft-tissue microcirculation need to be studied, as patients with proximal femur fractures often require such drugs.

## 5. Conclusions

To our knowledge, this is the first study to investigate the effects of traction on soft-tissue microcirculation in the treatment of trochanteric femur fractures. There were no changes in hemoglobin content, oxygen saturation, or blood flow before and after the application of traction. In conclusion, the use of a traction table in the treatment of proximal femur fractures does not seem to influence microcirculation.

## Figures and Tables

**Table 1:** Overview of mean values in arbitrary units (AU) with SD of the time points t0 and t1 in the three different depths and *p*-values of the one-way ANOVA.

	Depth (mm)	t0 (AU)	t1 (AU)	*p*-Value
SO_2_	2	13.33 ± 17.37	13.33 ± 16.62	*p* = 0.751
	8	65.00 ± 14.24	71.00 ± 17.24	*p* = 0.308
	15	64.67 ± 10.89	63.67 ± 12.05	*p* = 0.955
Hb	2	36.00 ± 9.95	34.67 ± 9.88	*p* = 0.651
	8	30.67 ± 8.47	29.33 ± 10.45	*p* = 0.928
	15	27.67 ± 7.55	28.83 ± 4.63	*p* = 0.926
Flow	2	11.83 ± 11.11	11.33 ± 10.85	*p* = 0.829
	8	96.00 ± 42.27	97.00 ± 46.95	*p* = 0.866
	15	173.00 ± 198.06	138.33 ± 64.93	*p* = 0.411

## Data Availability

Data are available from the authors upon reasonable request.
